# Human Serum Albumin Facilitates Heme-Iron Utilization by Fungi

**DOI:** 10.1128/mBio.00607-20

**Published:** 2020-04-21

**Authors:** Mariel Pinsky, Udita Roy, Shilat Moshe, Ziva Weissman, Daniel Kornitzer

**Affiliations:** a Department of Molecular Microbiology, B. Rappaport Faculty of Medicine, Technion—I.I.T. and the Rappaport Institute for Research in the Medical Sciences, Haifa, Israel; Institute of Molecular and Cell Biology; Tel Aviv University

**Keywords:** *Candida albicans*, heme transport, iron acquisition, mycology

## Abstract

A large portion of biological iron is found in the form of an iron-protoporphyrin IX complex, or heme. In the human host environment, which is exceptionally poor in free iron, heme iron, particularly from hemoglobin, constitutes a major source of iron for invading microbial pathogens. Several fungi were shown to utilize free heme, and Candida albicans, a major opportunistic pathogen, is able both to capture free heme and to extract heme from hemoglobin using a network of extracellular hemophores. Human serum albumin (HSA) is the most abundant host heme-scavenging protein. Tight binding of heme by HSA restricts its toxic chemical reactivity and could diminish its availability as an iron source for pathogenic microbes. We found, however, that rather than inhibiting heme utilization, HSA greatly increases availability of heme as an iron source for C. albicans and other fungi. In contrast, hemopexin, a low-abundance but high-affinity heme-scavenging serum protein, does inhibit heme utilization by C. albicans. However, inhibition by hemopexin is mitigated in the presence of HSA. Utilization of albumin-bound heme requires the same hemophore cascade as that which mediates hemoglobin-iron utilization. Accordingly, we found that the C. albicans hemophores are able to extract heme bound to HSA *in vitro*. Since many common drugs are known to bind to HSA, we tested whether they could interfere with heme-iron utilization. We show that utilization of albumin-bound heme by C. albicans can be inhibited by the anti-inflammatory drugs naproxen and salicylic acid.

## Abstract (precis)

Heme constitutes a major iron source for microorganisms and particularly for pathogenic microbes; to overcome the iron scarcity in the animal host, many pathogenic bacteria and fungi have developed systems to extract and take up heme from host proteins such as hemoglobin. Microbial heme uptake mechanisms are usually studied using growth media containing free heme or hemoglobin as a sole iron source. However, the animal host contains heme-scavenging proteins that could prevent this uptake. In the human host in particular, the most abundant serum heme-binding protein is albumin. Surprisingly, however, we found that in the case of fungi of the *Candida* species family, albumin promoted rather than prevented heme utilization. Albumin thus constitutes a human-specific factor that can affect heme-iron utilization and could serve as target for preventing heme-iron utilization by fungal pathogens. As a proof of principle, we identify two drugs that can inhibit albumin-stimulated heme utilization.

## INTRODUCTION

Iron is an essential element for most organisms. As a free element, or as part of iron-sulfur complexes or iron-protoporphyrin IX (heme), the ability of iron to switch between different oxidation states makes it a useful cofactor in biochemical catalysis. In the baker’s yeast Saccharomyces cerevisiae, almost 10% of all metabolic enzymes bind elemental iron or one of its complexes (1). However, the low solubility of the ferric ion, the main iron species present under oxidative conditions, makes iron acquisition challenging in most environments. Pathogenic microorganisms have additionally to contend with host iron-withholding mechanisms, part of so-called nutritional immunity, leading to extremely low free iron availability in the blood and tissues (2). Consequently, many pathogenic microbes have evolved systems to extract iron from host iron proteins (3).

Hemoglobin, the blood oxygen carrier protein, contains about 70% of the total body iron in humans. Pathogenic bacteria and fungi can utilize free heme as an iron source, and many can furthermore extract and utilize heme bound to hemoglobin (reviewed in references 4 and 5). Blood hemoglobin (normally around 150 mg/ml) is normally confined to the erythrocytes, but a certain level of hemolysis occurs normally, such that up to 0.25 mg/ml of free hemoglobin in serum is considered normal (6). Furthermore, many pathogens possess a hemolytic activity that can release additional hemoglobin to the serum. The released hemoglobin is captured by the serum protein haptoglobin, and the hemoglobin-haptoglobin complex is normally cleared by macrophages via the CD163 receptor (7). However, increased hemolysis, e.g., in the presence of microbial pathogens, can overwhelm the hemoglobin clearance capacity of haptoglobin. Free hemoglobin is rapidly oxidized to methemoglobin, which more readily releases its ferric heme, or hemin. Free hemin is ultimately captured by hemopexin, the serum protein with the highest affinity to heme, which delivers it to the liver for recycling or degradation (reviewed in reference 8). Albumin, the major human serum protein, also binds hemin, albeit at lower affinity than hemopexin; nonetheless, due to the 50-fold-higher molar concentration of human serum albumin (HSA) than of hemopexin, the majority of the released hemin is initially bound by albumin, and it is subsequently transferred to hemopexin only gradually (9).

The yeast Candida albicans is both a human commensal organism and an opportunistic pathogen, responsible for a large proportion of fungal systemic infections, particularly among immunocompromised individuals. Like many bacterial pathogens, C. albicans possesses a mechanism for the utilization of free hemin and hemoglobin-heme as iron sources (10, 11). The C. albicans heme utilization pathway relies on a family of soluble and extracellularly anchored hemophores, which bind heme via a CFEM domain (12). The soluble hemophore Csa2, the cell wall-anchored Rbt5, and the plasma membrane-anchored Pga7 all bind heme and extract it from hemoglobin and can transfer it among themselves, consistent with the notion that they form a heme transfer cascade across the cell envelope (12–14). Once at the plasma membrane, the hemin molecule is internalized to the vacuole by endocytosis (15).

Since albumin is the most abundant heme-binding protein in human serum, it could potentially prevent hemin utilization by invading pathogens. We therefore tested the effect of HSA on heme utilization by C. albicans and other fungi. However, we found that, rather than inhibiting heme uptake, HSA strongly promoted heme-iron utilization. We show that the hemoglobin-utilization CFEM hemophore cascade is required for heme-albumin utilization as well and further show that drugs that bind HSA can inhibit the albumin-stimulated heme-iron utilization.

## RESULTS

### Human serum albumin stimulates hemin and hemoglobin utilization.

**FIG 1:**
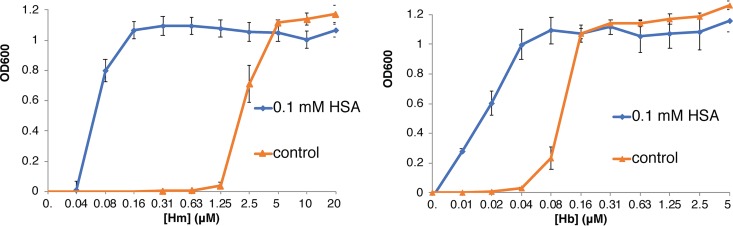
Stimulation of hemin (Hm) and hemoglobin (Hb) utilization by HSA. C. albicans *ccc2*^−/−^ cells (KC68), which are defective in high-affinity iron uptake and therefore do not grow in the presence of the iron chelator ferrozine, were incubated for 3 days at 30°C in YPD medium supplemented with 1 mM ferrozine, in increasing concentrations of hemin or hemoglobin, with or without the addition of 0.1 mM HSA, as indicated. Each culture was grown in triplicate; each point shows the average cell density, and the error bars indicate the standard deviations of the triplicate results. Hm = hemin, Hb = hemoglobin.

Utilization of hemin and hemoglobin by C. albicans is typically assayed in medium made to be iron-limiting by addition of the iron chelator ferrozine. While wild-type strains are able to grow in 1 mM ferrozine, growth of the C. albicans *ccc2*^−/−^ mutant, defective in high-affinity iron transport, is completely blocked in this medium and is therefore completely dependent upon alternative iron sources such as hemin or hemoglobin (11). Since albumin, the main human serum protein, was previously reported to bind hemin with an affinity of at least 10^−8^ M (16), we tested how addition of HSA would affect hemin and hemoglobin utilization in this assay. We found that addition of 0.1 mM HSA, instead of inhibiting heme utilization, lowered the hemin concentration required to achieve optimal growth by almost 2 orders of magnitude, whereas the hemoglobin concentration required was lowered 4-fold (Fig. 1). Bovine serum albumin (BSA) was previously reported to bind hemin less well than HSA (17) and to be less active than HSA in a heme transfer assay in cell culture (18); here also, we found that the ability of BSA to facilitate hemin and hemoglobin utilization by C. albicans is much lower than that of HSA (see Fig. S1 in the supplemental material).

10.1128/mBio.00607-20.1 FIG S1 BSA stimulates hemin utilization less well than HSA and does not stimulate hemoglobin utilization. C. albicans *ccc2*^−/−^ cells were grown as described for Fig. 1, in the presence of HSA and BSA, as indicated. Download FIG S1, JPG file, 0.8 MB. Copyright © 2020 Pinsky et al. 2020 Pinsky et al. This content is distributed under the terms of the Creative Commons Attribution 4.0 International license.

We next tried to identify the minimal concentration of HSA that is required for optimal facilitation of heme-iron utilization. Cells were grown with various albumin concentrations, with or without a fixed hemin concentration of 0.3 μM, or with fixed hemoglobin concentrations of 10 nM and 20 nM, previously shown to be insufficient for growth in the absence of HSA (Fig. 1). In the presence of 0.3 μM hemin, optimal growth was attained at around 3 μM HSA, but growth decreased sharply below 1 μM HSA (Fig. S2). In the presence of 10 nM or 20 nM hemoglobin, growth increased gradually with the HSA concentration up to the maximum tested (100 μM), with nonetheless a more moderate increase seen above 6 μM HSA (Fig. S2).

10.1128/mBio.00607-20.2 FIG S2 Stimulation of hemin and hemoglobin utilization with various HSA concentrations. C. albicans *ccc2*^−/−^ cells were grown as described for Fig. 1, with or without 0.3 μM hemin (left panel) or 10 nM or 20 nM hemoglobin (right panel), and with increasing amounts of HSA, as indicated. Download FIG S2, JPG file, 0.7 MB. Copyright © 2020 Pinsky et al. 2020 Pinsky et al. This content is distributed under the terms of the Creative Commons Attribution 4.0 International license.

To show that the stimulation of heme-iron utilization by HSA is not dependent on the type of chelator used, we repeated the experiment with 1 mM bathophenanthroline sulfonate (BPS), an alternative metal chelator that can also block growth of wild-type cells. In BPS-supplemented medium, wild-type and *ccc2*^−/−^ mutant cells had identical growth curves in increasing hemin or hemoglobin concentrations, in the presence or absence of HSA (Fig. S3).

10.1128/mBio.00607-20.3 FIG S3 HSA-mediated stimulation of hemin and hemoglobin utilization is independent of the iron chelator used and of the *CCC2* genotype. C. albicans strains wild type for *CCC2* (KC2) or *ccc2*^−/−^ (KC68) were grown as described for Fig. 1, with or without 0.1 mM HSA, except the YPD medium was supplemented with 1 mM of the iron chelator BPS, which inhibits growth of wild-type cells as well. Download FIG S3, JPG file, 1.1 MB. Copyright © 2020 Pinsky et al. 2020 Pinsky et al. This content is distributed under the terms of the Creative Commons Attribution 4.0 International license.

We conclude that albumin stimulation of hemin and hemoglobin utilization by C. albicans is dependent on human serum albumin specifically and is independent of the iron chelator used and of the high-affinity pathway of elemental iron uptake.

### The CFEM hemophore relay network is required for heme-albumin utilization.

Hemoglobin-iron utilization is completely dependent upon a set of closely related extracellular hemophores, Csa2, Rbt5, and Pga7, that capture heme from hemoglobin and transfer it across the cell envelope (12–14). These hemophores are not required to the same extent: the *csa2*^−/−^ mutant exhibits a weaker hemoglobin utilization phenotype, the *rbt5*^−/−^ mutant exhibits an intermediate phenotype, and the *pga7*^−/−^ mutant exhibits the strongest phenotype, being unable to utilize hemoglobin as an iron source. Utilization of free hemin is also defective in the absence of Rbt5 and Pga7, but, as described before (14), at a high enough hemin concentration, even the *pga7*^−/−^ mutant resumes growth, suggesting that free hemin can also enter the cell via a second, lower-affinity import system.

**FIG 2:**
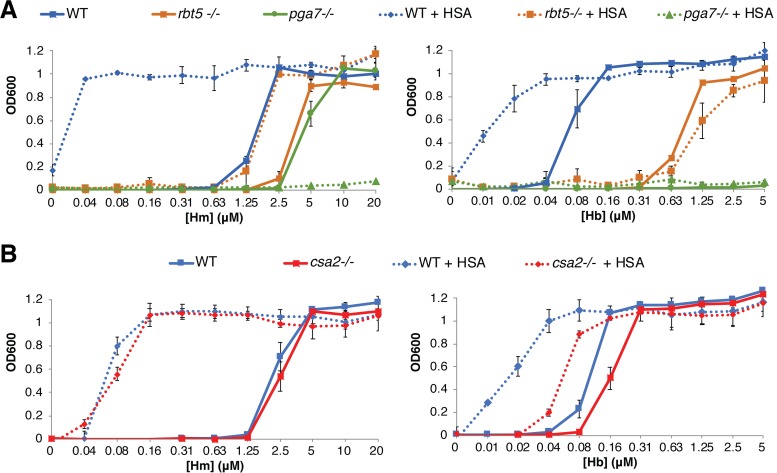
Heme-HSA utilization depends on the CFEM hemophore pathway. The C. albicans wild-type strain (KC68) and mutant strains *rbt5*^−/−^ (KC139), *pga7*^−/−^ (KC485), and *csa2*^−/−^ (KC782), all in the *ccc2*^−/−^ background, were grown as described for Fig. 1, with or without 0.1 mM HSA. (A) Mutants *rbt5*^−/−^ and *pga7*^−/−^ compared to the wild type. (B) Mutant *csa2*^−/−^ compared to the wild type.

We first tested the effect of HSA on these mutants with hemoglobin as the iron source. Addition of HSA to the medium could not rescue the growth of the *pga7*^−/−^ mutant and could not improve the residual growth of the *rbt5*^−/−^ mutant in hemoglobin (Fig. 2A). Addition of HSA, however, still considerably improved hemoglobin utilization in the *csa2*^−/−^ mutant, to the extent that this mutant, in the presence of HSA, required less hemoglobin than the wild-type strain in the absence of HSA (Fig. 2B).

In hemin as the sole iron source, the *csa2*^−/−^ mutant showed no growth defect in the absence of HSA, and addition of HSA improved hemin utilization to the same extent as in the wild-type strain (Fig. 2B). Addition of HSA slightly improved growth of the *rbt5*^−/−^ mutant, but interestingly, for the *pga7*^−/−^ mutant, which is able to grow in higher hemin concentrations, addition of HSA abolished this residual growth (Fig. 2A). The latter result indicates that utilization of albumin-bound hemin is entirely dependent upon the CFEM hemophore cascade, as in the absence of Pga7, the most essential member of the heme transfer network, HSA becomes inhibitory to free hemin utilization, presumably by binding to it and preventing its uptake by an alternative pathway.

### CFEM proteins extract heme from HSA.

**FIG 3:**
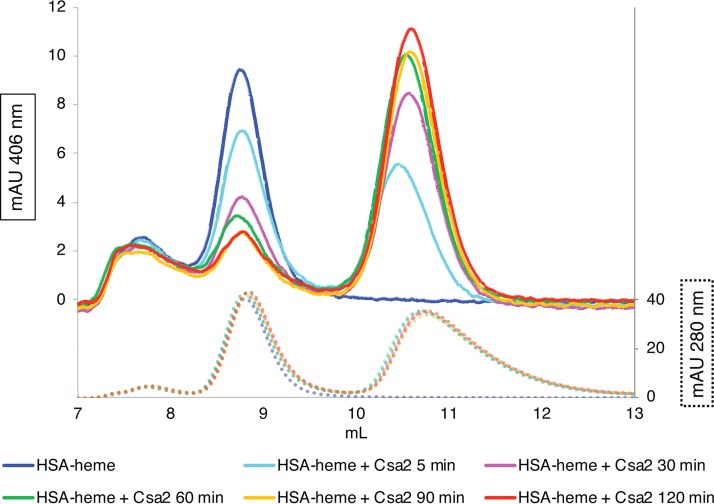
The CFEM hemophore Csa2 can extract heme from HSA. HSA samples (25 μl of 25 μM) preloaded with 10 μM hemin were mixed with 25 μl of 25 μM Csa2, incubated at room temperature for the indicated amount of time, and then loaded on a SEC column. The graph indicates the migration of heme (top; detected at 406 nm; solid lines) versus migration of total protein (bottom; detected at 280 nm; dotted lines) on the column. Retention volumes of HSA and Csa2 are indicated. Color coding is the same for the 406-nm and 280-nm curves. The 280-nm curves indicate that the total amount of protein did not vary throughout the experiment. mAU, milli-absorbance units.

Since the CFEM protein cascade is required for utilization of hemin bound to HSA, we tested whether the CFEM hemophores can extract hemin from HSA *in vitro*. We first used size exclusion chromatography (SEC) to follow heme bound to HSA versus to the soluble hemophore Csa2 (the migration of the other CFEM hemophores is too similar to that of HSA to allow separation by SEC). Differential migration of Csa2 and HSA on SEC, coupled with the ability to measure the amount of heme bound to each protein based on its absorbance in the visible part of the spectrum, enabled following the migration of the heme from HSA to Csa2. The proteins were mixed and then incubated for various incubation times and loaded onto a SEC column for separation. The shortest incubation time, including the time to load the column and the time to achieve sufficient initial separation between the proteins, is roughly estimated to be 5 to 10 min. As shown in Fig. 3, even after this short incubation, about half the heme had migrated from HSA to Csa2. After 60 min of incubation, the vast majority of the heme had moved to Csa2. In contrast, we found that the Csa2 D80A mutant, which lacks the heme iron-coordinating Asp80 (12) and is therefore unable to bind heme, was unable to release heme from HSA (Fig. S4).

10.1128/mBio.00607-20.4 FIG S4 The Csa2 D80A mutant is unable to extract heme from HSA. The experiment was performed similarly to the experiment whose results are shown in Fig. 3, except that the concentrations of protein (50 μM) and of heme (20 μM) that were loaded were doubled and the Csa2 D80A mutant, defective in heme binding, was used. Download FIG S4, JPG file, 0.9 MB. Copyright © 2020 Pinsky et al. 2020 Pinsky et al. This content is distributed under the terms of the Creative Commons Attribution 4.0 International license.

**FIG 4:**
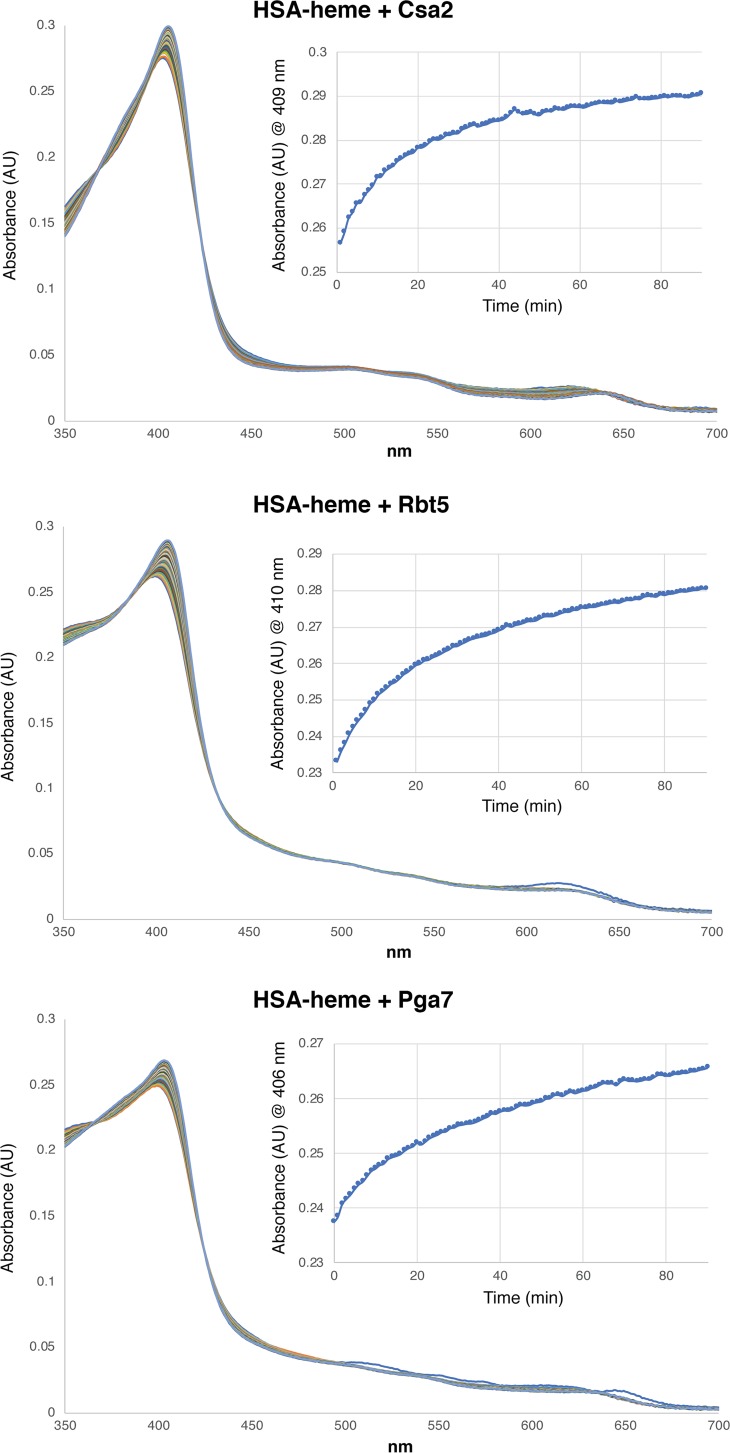
All three CFEM hemophores extract heme from HSA. HSA (10 μM) was preloaded with 5 μM hemin in a spectroscopy cuvette, and then the indicated CFEM protein was added to 10 μM, and absorption spectra were taken every minute for 90 min. The inset shows the change in absorbance with time, at the wavelength that distinguishes best between heme bound to HSA and heme bound to the relevant CFEM protein.

A second method for measuring transfer of heme from HSA to CFEM proteins was based on the distinct spectral properties of heme bound to the different proteins. HSA-heme gives a maximum absorbance (“Soret peak”) at 403 nm, whereas the CFEM protein-heme complexes absorb maximally at 406 nm. Other absorbance peaks also differ between the proteins, notably in the 620-nm to 640-nm range, but the largest difference is seen between 406 and 410 nm, depending on the proteins that are compared. Spectroscopy allowed us to monitor heme transfer from HSA to Pga7 and to Rbt5, proteins that cannot be easily separated from HSA by SEC, and also allowed us to achieve much better time resolution. As shown in Fig. 4, the transfer kinetics between HSA and Csa2 are similar to those determined by SEC, with a plateau being reached after an hour, and a half-maximal saturation of Csa2 being obtained after about 10 min. The HSA-Rbt5 and HSA-Pga7 heme transfer kinetics were somewhat slower, with half-maximal saturation being obtained after about 17 min.

### Additional *Candida* species can utilize hemoglobin and HSA-heme.

**FIG 5:**
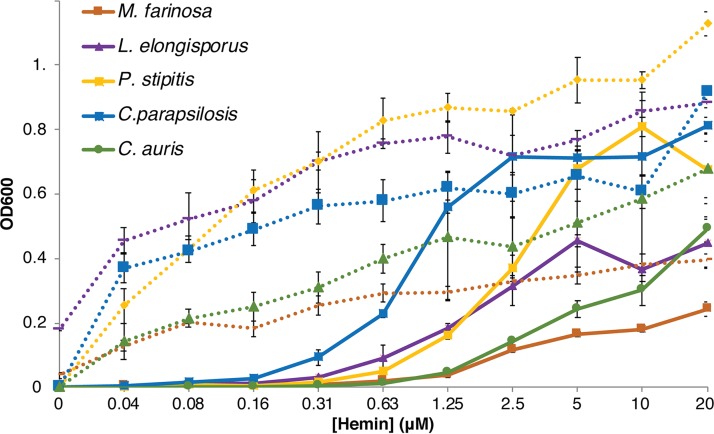
Utilization of hemin and stimulation by HSA in additional species. The indicated species were grown as described for Fig. 1, in YPD medium supplemented with 1 mM BPS. The full lines indicate growth in the absence of HSA, and the dotted lines indicate growth with 0.1 mM HSA supplementation. The line color is the same for each species with and without HSA.

Many additional *Candida* species have genes encoding CFEM hemophore homologs, but only C. parapsilosis was previously shown to be able to utilize heme as an iron source (19), and no information was available regarding hemoglobin utilization. We first tested hemoglobin utilization in five species of the Debaryomycetaceae clade that includes *Candida* spp., using YPD medium (1% yeast extract, 2% Bacto peptone, 2% glucose, tryptophan 150 mg/liter) supplemented with the iron chelator BPS. All five species were able to grow on hemoglobin as an iron source (Fig. S5). We then tested both utilization of hemin and its stimulation by HSA. As shown in Fig. 5, all five species—C. parapsilosis, C. auris, Lodderomyces elongisporus, *Pichia* (*Scheffersomyces*) *stipitis*, and Millerozyma farinosa—could utilize hemin, but in the presence of HSA, lower hemin concentrations sufficed to obtain the same growth. We conclude that utilization of hemoglobin-heme and HSA-heme is not specific to C. albicans or other commensal species such as C. parapsilosis but is exhibited by a wide spectrum of *Candida* species that express CFEM hemophore homologs.

10.1128/mBio.00607-20.5 FIG S5 Utilization of hemoglobin iron by additional fungal species. The indicated species were grown as described for Fig. 1, in YPD medium–1 mM BPS. Download FIG S5, JPG file, 0.6 MB. Copyright © 2020 Pinsky et al. 2020 Pinsky et al. This content is distributed under the terms of the Creative Commons Attribution 4.0 International license.

### Effect of hemopexin on heme utilization.

Hemopexin is the serum heme-binding protein that has the highest affinity for heme, with an estimated dissociation constant (*K_d_*) of 10^−13^ M (20), enabling it to extract heme from HSA (9). Binding of heme to hemopexin and binding to HSA can be differentiated by the different wavelengths of their respective maximal absorbance peaks (Fig. S6A). When hemopexin was mixed with HSA at a 1:50 ratio, which is similar to their ratio in serum, initial absorbance indicated binding mainly to HSA, and over a 12-h period, heme gradually shifted to binding to hemopexin (Fig. S6B). These results are similar to those obtained previously by Morgan et al. (9).

10.1128/mBio.00607-20.6 FIG S6 Hemopexin extracts heme from albumin. (A) Absorbance spectra of 2 μM heme mixed with 150 μM HSA, with 3 μM hemopexin, or with 150 μM HSA–3 μM hemopexin, all in PBS buffer at room temperature. Spectra around the Soret maximum were recorded continually for 1 h (HSA, hemopexin) or for 24 h (HSA plus hemopexin). Only the absorbance spectra present at the beginning and at the end of the incubations are depicted. (B) The ratio between the absorbance at 413 nm (hemopexin) and the absorbance at 403 nm (HSA) was plotted against time. The absorbance ratios of heme mixed with hemopexin or HSA stabilized within 10 min. The absorbance ratio of heme added to the protein mixture indicates that heme bound initially to HSA and gradually migrated to hemopexin over 12 h. Download FIG S6, JPG file, 1.0 MB. Copyright © 2020 Pinsky et al. 2020 Pinsky et al. This content is distributed under the terms of the Creative Commons Attribution 4.0 International license.

**FIG 6:**
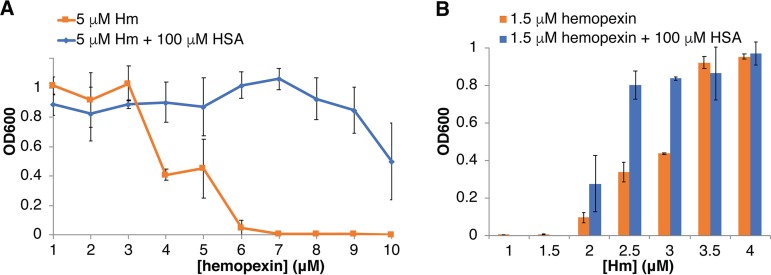
HSA mitigates hemopexin inhibition of heme utilization by C. albicans. (A) C. albicans *ccc2*^−/−^ cells were grown as described for Fig. 1 for 2 days, in the presence of 5 μM hemin, with or without 100 μM HSA as indicated, in increasing human hemopexin concentrations. (B) Cells were grown 3 days in the presence of 1.5 μM hemopexin, with or without 100 μM HSA as indicated, in increasing hemin concentrations.

We next tested the effect of hemopexin on hemin utilization by C. albicans. We found that with a fixed hemin concentration of 5 μM, hemopexin inhibited growth at equimolar or higher concentrations, suggesting that C. albicans is unable to utilize heme bound to hemopexin (Fig. 6A). Consistent with this, we found that the C. albicans hemophore Csa2 was unable to extract heme from hemopexin *in vitro* (data not shown). Addition of 100 μM HSA, however, greatly mitigated this inhibition (Fig. 6A). In a second experiment, we tested the effect of a fixed 1.5 μM hemopexin concentration on growth with increasing hemin concentrations. Growth was inhibited in 1.5 μM hemin or lower, even in the presence of HSA, but at intermediate hemin concentrations, growth was increased in the presence of HSA (Fig. S6B). We conclude that while hemopexin is able to inhibit heme utilization by C. albicans, the presence of HSA can mitigate this effect.

### Effect of HSA-binding drugs on hemin utilization and transfer.

Many commonly used drugs are known to bind to HSA (reviewed in reference 21), and several of these drugs were previously shown to allosterically interfere with hemin binding to HSA. In order to test drug effects on HSA-facilitated heme utilization, a hemin concentration (0.3 μM) that did not support growth in the absence of HSA was used, together with 5 μM HSA. As controls for possible effects on HSA-independent heme and iron utilization, we used 3 μM hemin in the absence of HSA, as well as the *CCC2^+/+^* strain, which does not require heme as an iron source under the test conditions.

**FIG 7:**
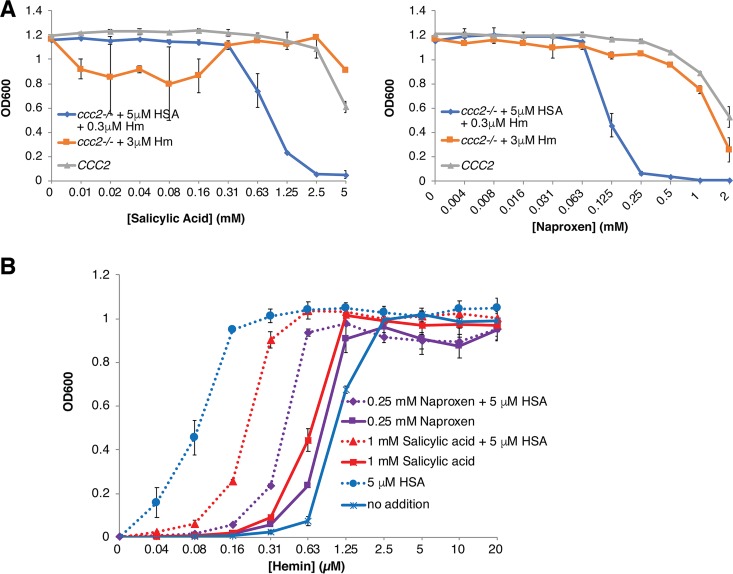
Salicylic acid and naproxen inhibit HSA-stimulated heme utilization. (A) The *CCC2^+/+^* strain (KC2) and the *ccc2*^−/−^ strain (KC68) supplemented with 3 μM heme or 0.3 μM heme–5 μM HSA were grown as described for Fig. 1, in increasing concentrations of salicylic acid or of naproxen. (B) The *ccc2*^−/−^ strain was grown with or without 0.25 mM naproxen or 1 mM salicylic acid, and with or without 5 μM HSA, in increasing concentrations of hemin.

Of four drugs (isoniazid, rifampin, warfarin, and ibuprofen) previously shown to allosterically interfere with heme binding to HSA (22–24) that were tested, none affected HSA-facilitated hemin utilization by C. albicans (see Table S1 in the supplemental material). We next tested four additional drugs—salicylic acid, naproxen, camptothecin, and fusidic acid—the binding sites of which overlap the heme binding site on HSA (25–27) (Fig. S7). While camptothecin showed no effect at up to 0.15 mM and fusidic acid at up to 2 mM (Table S1), both naproxen and salicylic acid started interfering with HSA-facilitated heme utilization at 0.13 mM (naproxen) and 1 mM (salicylic acid) (Fig. 7A). In a second experiment, the effect of 1 mM salicylic acid or 0.25 mM naproxen was tested with various hemin concentrations, with or without 5 μM HSA. As shown in Fig. 7B, these drug concentrations clearly reduced, but did not completely cancel, the HSA-induced stimulation of hemin utilization. Utilization of 0.3 μM hemin in the presence of 1 mM salicylic acid or 0.13 mM naproxen was also recovered when HSA concentration was increased to 25 to 50 μM (Fig. S8), indicating that higher HSA concentrations can overcome inhibition by these drugs.

10.1128/mBio.00607-20.7 FIG S7 Alignment of heme bound to HSA with four HSA-binding drugs. The crystal structure of HSA bound to heme (PDB accession no. 1N5U; the heme is colored purple) was aligned with that of the following drugs bound to HSA: salicylic acid (3B9M; green), naproxen (2VDB; red), camptothecin (4L9K; yellow), and fusidic acid (2VUF; blue). The trace of the HSA backbone between positions 110 and 195 (PDB 1N5U) is shown in gray. The alignments were done by the use of Chimera. Download FIG S7, JPG file, 2.7 MB. Copyright © 2020 Pinsky et al. 2020 Pinsky et al. This content is distributed under the terms of the Creative Commons Attribution 4.0 International license.

10.1128/mBio.00607-20.8 FIG S8 Higher HSA concentrations can overcome inhibition of hemin utilization by salicylic acid and naproxen. KC68 cells were grown as described for Fig. 1, with 0.3 μM hemin, without or with 1 mM salicylic acid (SA; left panel) or 0.25 mM naproxen (right panel), in increasing concentrations of HSA, as indicated. Download FIG S8, JPG file, 0.7 MB. Copyright © 2020 Pinsky et al. 2020 Pinsky et al. This content is distributed under the terms of the Creative Commons Attribution 4.0 International license.

10.1128/mBio.00607-20.9 TABLE S1 Summary of the effect of HSA-binding drugs on C. albicans growth and on heme utilization stimulated by 5 μM HSA. Growth was tested with the C. albicans KC2 (*CCC2*) strain in YPD medium–1 mM ferrozine and with the KC68 (*ccc2*^−/−^) strain in the same medium supplemented with 3 μM hemin. HSA-heme utilization was tested in the KC68 background, in the same medium supplemented with 5 μM HSA and 0.3 μM hemin. Download Table S1, PDF file, 0.08 MB. Copyright © 2020 Pinsky et al. 2020 Pinsky et al. This content is distributed under the terms of the Creative Commons Attribution 4.0 International license.

**FIG 8:**
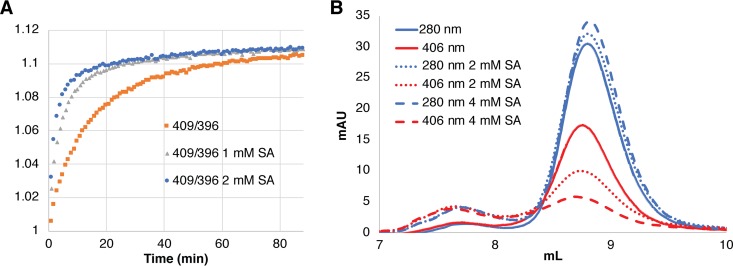
Effect of salicylic acid on heme transfer and binding. (A) The different ratios of absorbances at 409 nm and 396 nm between heme bound to HSA and heme to Csa2 were used to monitor transfer of heme from HSA to Csa2. HSA (10 μM) was preloaded with 5 μM heme in regular PBS buffer or in buffer supplemented with 1 mM or 2 mM salicylic acid (SA). (B) HSA (20 μM) was incubated 5 min with 10 μM hemin in 50 μl PBS buffer, without or with 2 mM or 4 mM SA, and then loaded on a 24-ml SEC column loaded with PBS buffer or preloaded with a 5 ml layer of PBS–2 mM SA or PBS–4 mM SA, respectively (the high 280-nm absorbance level of SA precluded loading the whole column with PBS plus SA). The HSA protein peak was detected by monitoring absorbance at 280 nm, and the heme bound to HSA was detected at 406 nm.

In order to test whether the effect of these two drugs is due to inhibition of heme binding to HSA or to inhibition of heme transfer from HSA to the CFEM proteins, we attempted to analyze their effect on heme binding and transfer *in vitro*. Because the low solubility of naproxen at relevant concentrations hampered spectroscopic analysis, we focused on salicylic acid. By spectroscopy, addition of up to 4 mM salicylic acid did not appear to inhibit heme binding to HSA (not shown). We then tested the effect of salicylic acid on heme transfer from HSA to Csa2, by relying on the different levels of absorbance of heme bound to HSA versus heme bound to Csa2 at 409 nm, normalized to the invariant absorbance at 396 nm. When heme transfer between HSA and Csa2 was monitored in 1 mM or 2 mM salicylic acid, the transfer was actually faster than in control buffer without salicylic acid, with half-maximal transfer attained within 3 min (2 mM) and 5 min (1 mM), versus 10 min for the control buffer (Fig. 8A). This result suggested that salicylic acid interferes with heme binding to HSA enough to facilitate its release to Csa2 but not enough to prevent binding of free hemin by HSA.

In order to test the effect of salicylic acid on hemin binding to HSA more directly, we migrated HSA-heme through an SEC column preloaded with a layer of buffer containing salicylic acid. As shown in Fig. 8B, migration through 2 mM or 4 mM salicylic acid led to gradual removal of hemin from the HSA, consistent with competition of this compound with hemin for binding to HSA. We thus conclude that the effect of salicylic acid is due to competition with heme for binding to HSA, rather than to inhibition of transfer of the heme from HSA to the CFEM proteins.

**FIG 9:**
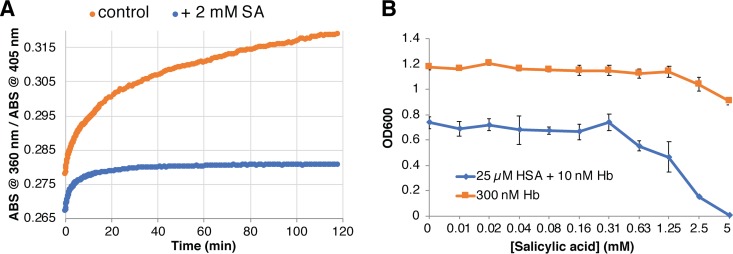
Salicylic acid inhibits heme transfer from hemoglobin to HSA and HSA-stimulated hemoglobin utilization. (A) The different ratios of absorbances (ABS) at 405 nm and 360 nm between heme bound to HSA and heme bound to globin were used to monitor transfer of heme from hemoglobin to HSA. Hemoglobin (2.5 μM) was mixed with 20 μM HSA in regular PBS buffer or in buffer supplemented with 2 mM salicylic acid (SA). (B) C. albicans *ccc2*^−/−^ cells (KC68) were grown as described for Fig. 1 either in 10 nM hemoglobin (Hb) with 25 μM HSA or in 0.3 μM hemoglobin, and in increasing concentrations of salicylic acid. Note that growth saturation was attained at a lower density in 10 nM hemoglobin, probably due to the limiting iron concentration.

Albumin was also shown to be able to capture heme from hemoglobin (17). We followed the transfer of heme from globin to HSA by spectroscopy, relying on the different spectroscopic properties of heme bound to globin versus to albumin. We found that this transfer was almost completely inhibited by salicylic acid (Fig. 9A). We then tested whether salicylic acid could inhibit the HSA-stimulated hemoglobin-heme utilization as well. Since the stimulation of hemoglobin-heme utilization is best seen at very low hemoglobin concentrations (Fig. 1; see also Fig. S1 and S2), we used 10 nM hemoglobin and 25 μM HSA and tested the effect of increasing concentrations of salicylic acid on growth. As shown in Fig. 9B, growth was gradually inhibited at 0.6 mM salicylic acid and above, whereas the effect of salicylic acid on the control culture grown in 0.3 μM hemoglobin remained minimal.

## DISCUSSION

In order to analyze the complex web of interactions between host and pathogen, it is usually broken down into single interactions that can be studied individually *in vitro*. With regard to the pathogen function studied here, namely, acquisition of host heme iron, analysis is typically performed in culture by feeding the microbes with hemin or hemoglobin in regular growth medium depleted of free iron. The danger of this approach is that the contribution of additional important host factors to the interaction being studied can be easily overlooked. Here, we show that a major host heme-binding protein, albumin, plays a potentially important role in heme-iron and hemoglobin-iron acquisition by pathogenic fungi.

Human albumin binds heme with high affinity and is thought to function as an inhibitor of free heme toxicity in serum. Potentially, heme binding by HSA could also prevent the ability of microorganisms to utilize heme as an iron source. Nonetheless there are reports that some bacteria, including both Gram-positive and -negative species, are able to utilize albumin-bound heme as heme and iron sources (28, 29). Here, we have shown that fungi are likewise able to utilize HSA-bound hemin as an iron source in addition to free heme and to hemoglobin. Furthermore, not only does addition of HSA not inhibit heme-iron utilization, but it makes it more efficient, as the free hemin and, to a lesser measure, the hemoglobin concentrations required for growth are drastically lowered in the presence of HSA. Furthermore, stimulation of heme-iron utilization was seen at concentrations as low as 5 μM HSA, i.e., over 2 orders of magnitude lower than the serum albumin concentration. Thus, albumin leaking from the serum into tissues (the concentration of interstitial fluid albumin was found to be in the 65 to 200 μM range, representing 1/10 to 1/3 its concentration in serum [30, 31]) could be a significant factor in heme-iron acquisition for fungi that have left the bloodstream and penetrated tissues.

The stimulatory activity of HSA in heme-iron utilization could be explained, in the case of free heme, by the tendency of the heme molecules to aggregate in the form of stacked arrays in solution, which reduces the effective concentration of heme in the medium. The effect of HSA could be to resolubilize these aggregates, thereby increasing the effective heme concentration available to the microorganisms. For hemoglobin heme utilization, where a significant stimulation by HSA was also detected, the role of HSA is less clear. We note, however, that HSA was reported to be able to capture heme released from methemoglobin (Fe^3+^ hemoglobin) (17) (Fig. 9). It is possible that heme albumin is a more readily available heme source for the fungi than methemoglobin.

**FIG 10:**
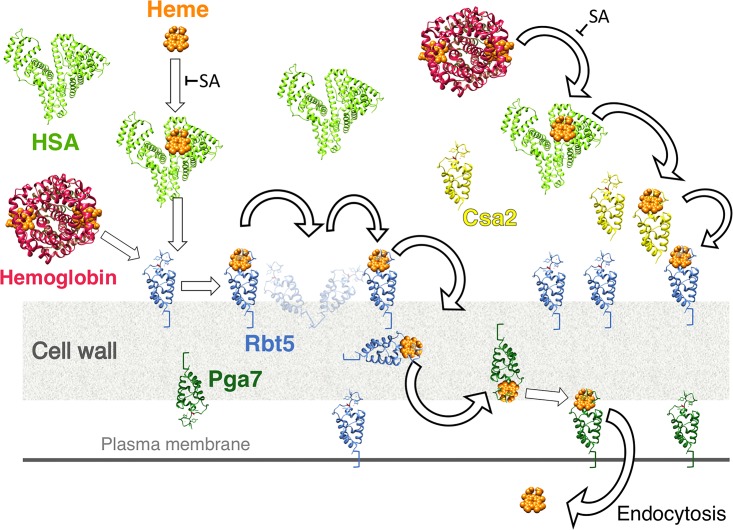
Model for the role of human serum albumin in the acquisition of heme by pathogenic fungi. CFEM domain proteins Csa2, Rbt5, and Pga7 are depicted in yellow, blue, and dark green, respectively; hemoglobin is indicated in red and HSA in light green. SA = salicylic acid.

The fungal system responsible for utilization of heme bound to HSA is the same as that used for hemoglobin-heme utilization, namely, the extracellular CFEM hemophore cascade system, which consists of a secreted hemophore, Csa2, a cell wall-peripheral hemophore, Rbt5, and a membrane-proximal hemophore, Pga7. Respective deletions of the three corresponding genes show an increasing defect of hemin and hemoglobin utilization (12). The most telling phenotype with regard to heme-HSA utilization is that of the *pga7*^−/−^ mutant, which is essential for hemoglobin utilization but can be partially bypassed when free hemin is the iron source, indicating the existence of an independent pathway for free hemin uptake (14). The paradoxical observation that whereas HSA made hemin more available for wild-type cells, it made it less available for the *pga7*^−/−^ mutant (Fig. 2A), can be explained if the CFEM hemophore pathway is required for heme-albumin utilization. In support of this model, we reproduced heme extraction from HSA *in vitro* with all three CFEM hemophores. Thus, the pathway of heme uptake into C. albicans and related fungi must be updated to include, in the host context, albumin as an intermediate receptor between free heme or hemoglobin and the CFEM cascade (Fig. 10).

The mutant lacking the soluble hemophore Csa2 is mildly defective in hemoglobin utilization and shows no defect in hemin utilization. The presence of HSA greatly improved hemoglobin utilization in the mutant, but it was still defective compared to that of the wild-type strain with HSA, whereas with hemin as an iron source, the level of HSA stimulation seen with the *csa2*^−/−^ mutant was equal to that seen with the wild type (Fig. 2B). The effect of HSA on the mutant lacking the third CFEM hemophore, the peripheral cell wall protein Rbt5, was more complex: the addition of HSA did not seem to improve the hemoglobin utilization of the *rbt5*^−/−^ mutant and may have even slightly reduced it, whereas it still improved hemin utilization by this mutant (Fig. 2A). The phenotypes of the *csa2*^−/−^ and *rbt5*^−/−^ mutants might reflect a complex pathway of heme transfer between hemoglobin, albumin, and the CFEM hemophores.

The mechanism for heme transfer from HSA to the CFEM proteins could involve direct interaction between the proteins or capture by the CFEM proteins of heme released by the albumin without direct interaction. C. albicans cells induced to make hyphae, which usually also express CFEM hemophores, including the abundant cell wall protein Rbt5 (5), were previously reported to bind albumin (32), suggesting the possibility of a direct interaction. With the soluble hemophore Csa2, SEC migration does not suggest interaction with HSA, but the possibility of a transient, low-affinity interaction cannot be excluded. An additional possible mechanism for the release of heme from host proteins is proteolytic degradation of the protein by secreted fungal proteases. The secreted aspartic protease Sap2 is induced when albumin is the sole nitrogen source in the medium (33) and is required for utilization of albumin as nitrogen source (34). The large amount of HSA and low molar ratio of heme-HSA in serum would, however, make this an inefficient mechanism, and furthermore unnecessary, since we found that the CFEM hemophores can capture heme from HSA. Proteolytic degradation might, however, be a potential mechanism for extracting heme from hemopexin in nitrogen-limiting medium.

Hemopexin is a serum heme-scavenging protein conserved among all vertebrates and has a higher affinity to heme than HSA (35). In spite of its strong affinity for heme, some bacterial pathogens express proteins that can dislodge heme from hemopexin (36, 37), while others can overcome hemopexin inhibition of heme-iron utilization only by degrading it with secreted proteases (38). Here, we show that hemopexin does inhibit heme-iron utilization by C. albicans up to an equimolar concentration of heme but not above that. Thus, given the low serum concentration of hemopexin, under conditions of increased hemolysis induced, e.g., by a hemolytic function of C. albicans (39), its limited heme buffering capacity could be easily overwhelmed. In addition, we found that inhibition of heme utilization by hemopexin is mitigated by the presence of HSA. Finally, heme released to a 1:50 hemopexin-HSA mixture is first bound by HSA before being gradually transferred to hemopexin. Thus, in the dynamic blood environment where heme is continuously released to the medium by hemolysis, at any given time a significant fraction of heme could be found in HSA, where it would be available to the C. albicans CFEM hemophore cascade.

Heme-binding properties are reported to differ between human serum albumin and albumin from other species. We found that bovine serum albumin is much less active than HSA in promoting heme utilization. This is in accordance with functional studies that showed much better heme transfer from hemoglobin to primate albumin than to albumin from other sources (17) and reduced uptake of heme by animal cells in the presence of BSA versus HSA (18). Structural studies of different albumins also suggested that the heme binding site is specific to primate serum albumin (40, 41). This heme specificity of the human albumin suggests that animal model systems might be a poor substitute for testing the role of heme uptake in the virulence of human pathogens in general and of pathogenic fungi in particular.

Many common drugs are known to bind HSA (21), and some of them were previously shown to allosterically inhibit heme binding to HSA (23, 24). However, none of the four allosterically binding drugs tested—warfarin, ibuprofen, isoniazid, and rifampin—were found to inhibit HSA-stimulated heme utilization. We therefore analyzed the crystal structure of HSA bound by additional drugs and detected four molecules with binding sites overlapping that of the heme: salicylic acid (42), naproxen (26), camptothecin (25), and fusidic acid (43) (see Fig. S7 in the supplemental material). The first two showed clear inhibition of HSA-stimulated heme utilization at concentrations below toxic levels.

The two drugs that were found to inhibit the HSA-mediated stimulation of heme utilization, salicylic acid and naproxen, did so at concentrations corresponding to therapeutic serum concentrations in patients (44), suggesting that these drugs could be relevant *in vivo*. However, this range of drug concentrations was inhibitory only when the HSA concentration was kept low; at HSA concentrations closer to serum concentrations, the drug dosage was not sufficient to inhibit heme utilization. It is nonetheless possible that in tissues, where albumin and hemoglobin concentrations are lower, these drugs could be relevant to inhibition of heme-iron utilization by fungal pathogens.

To conclude, we have shown that a major human serum protein, HSA, could play an important role in facilitating heme-iron utilization by fungal pathogens. Our results suggest that HSA can in principle be pharmacologically targeted in order to inhibit heme-iron utilization by fungi but that molecules with higher affinity to the HSA heme-binding site than salicylic acid or naproxen might be required. Importantly, heme and hemoglobin serve as iron sources for many bacterial pathogens as well (45, 46). While heme bound to albumin was shown in a few cases to be available to bacteria (28, 29), the ability of HSA to generally facilitate heme-iron utilization by bacterial pathogens remains to be tested.

## MATERIALS AND METHODS

### Strains and materials.

Fungal strains used are listed in Table S2 in the supplemental material. Cells were grown throughout in YPD medium (1% yeast extract, 2% Bacto peptone, 2% glucose, tryptophan 150 mg/liter) supplemented with an ion chelator (ferrozine or bathophenanthroline sulfonate; Sigma) at 1 mM and hemin from a 2 mM stock in 50 mM NaOH or hemoglobin from a 0.5 mM stock in phosphate-buffered saline (PBS; 137 mM NaCl, 2.7 mM KCl, 10 mM Na_2_HPO_4_, 2 mM KH_2_PO_4_, pH = 7.4). Hemin was obtained from Frontier Scientific and HSA (A3782), BSA (A3912), and bovine hemoglobin (H2500) from Sigma. Naproxen, isoniazid, rifampin, ibuprofen, warfarin, and camptothecin were obtained from Sigma; salicylic acid from Alpha Aesar; and fusidic acid from GoldBio. The Csa2, Pga7, and Rbt5 wild-type proteins were produced in Pichia pastoris as described in references 12 and 14. The Csa2 D80A mutant was produced similarly, using P. pastoris transformed with plasmid KB2411. KB2411 was derived from KB2366 (12) by site-directed mutagenesis.

10.1128/mBio.00607-20.10 TABLE S2 List of fungal strains. Download Table S2, PDF file, 0.08 MB. Copyright © 2020 Pinsky et al. 2020 Pinsky et al. This content is distributed under the terms of the Creative Commons Attribution 4.0 International license.

Hemopexin was purified from human serum (Sigma H4522) or from expired human plasma obtained as research material from the Magen David Adom blood bank. Use of human plasma from donors was authorized by the Technion’s Institutional Review Board (approval no. 13345). The purification protocol, based on methods described in reference 47, was as follows: a 2 ml hemin-agarose column (Sigma H6390) placed in NP buffer (0.5 M NaCl, 10 mM NaPO_4_, pH 7.5) was loaded with 50 ml of serum or plasma, washed with 20 volumes of NP buffer, and then eluted with 5 ml 0.2 M sodium citrate (pH 3.8 to 4.0). The eluate was immediately neutralized with 0.5 ml 1 M Tris (pH 7.5)–0.09 ml NaOH 10 N. A single 50-ml serum or plasma volume could be passaged 3 or 4 times over the column before it was depleted of hemopexin, yielding up to 20 mg of protein in total. Eluates were combined, concentrated on an Amicon-30-kDa-cutoff concentrator, and washed and resuspended in PBS. Purity was determined by SDS-PAGE to be 90%. Heme binding activity was determined by spectrophotometric detection of the expected 413-nm Soret absorbance peak (48). Since we found that freezing and thawing greatly reduced activity, hemopexin batches were kept at 4°C and used within a few days. The hemopexin purified from purchased human serum and that from donor plasma had similar activity levels and gave similar results.

### Growth assays.

Overnight cultures grown in YPD medium were diluted in the morning into a series of 2-fold dilutions of hemin or hemoglobin, or of HSA or drug, as indicated, in YPD medium plus ferrozine or BPS. Cells were inoculated in ﬂat-bottomed 96-well plates at 150 μl per well at an optical density at 600 nm (OD_600_) of 0.00001. Plates were incubated at 30°C on an orbital shaker at 60 rpm, and growth was measured by OD_600_ after 2 and 3 days with an enzyme-linked immunosorbent assay (ELISA) reader. Cells were resuspended with a multipipettor before each reading. Each culture procedure was done in triplicate.

### Size exclusion chromatography.

Samples (50 μl) were injected into a Superdex 75 10/300 column (GE Healthcare) equilibrated with PBS and were monitored on an AKTA Purifier system. Absorbance was recorded at 280 nm, 380 nm, and 406 nm. For migration through salicylic acid, the column was preloaded with 5 ml (of a 20-ml column volume) of PBS–salicylic acid 2 mM or 4 mM, prior to loading of the sample. All experiments were done at room temperature.

### UV/VIS spectroscopy.

Absorption spectra were recorded using a Cary 60 spectrophotometer with a 1-ml, 10-mm-optical-path quartz cuvette. Kinetics of absorbance were obtained with Cary scanning kinetics software and were processed by the use of Microsoft Excel.
