# Supplementary figures and images for: Human Serum Albumin Facilitates Heme-Iron Utilization by Fungi
Source: mBio. 2020 Apr 21;11(2):e00607-20. doi: 10.1128/mBio.00607-20 (PMC7175094; doi:10.1128/mBio.00607-20)

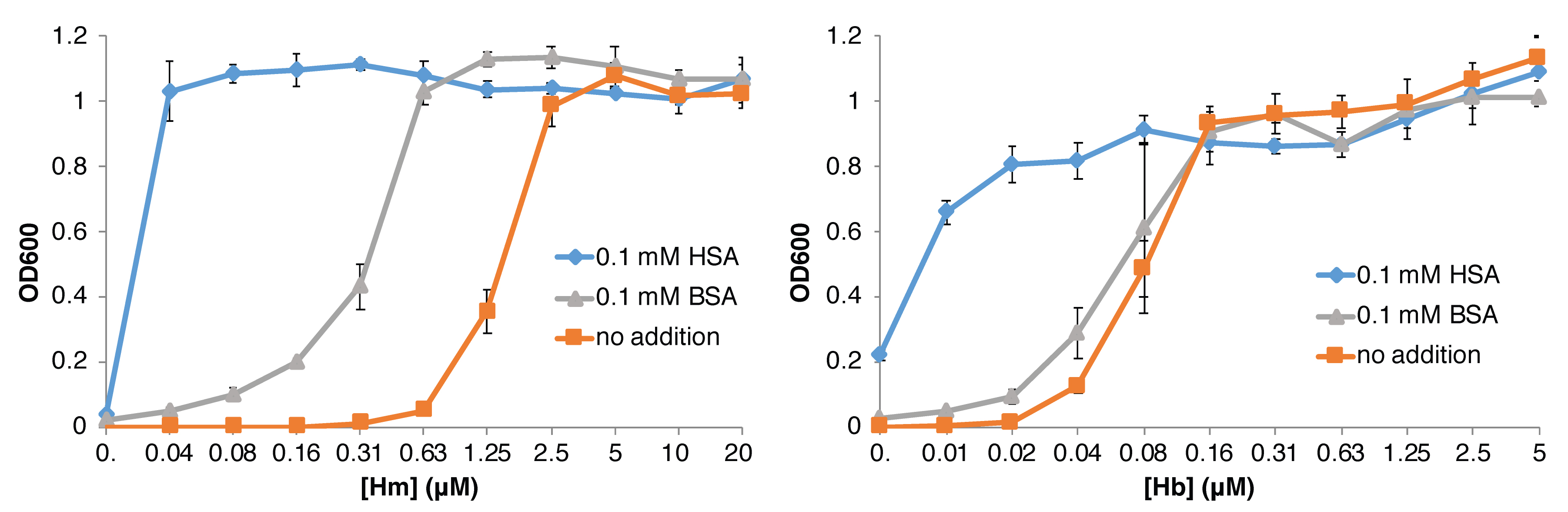

Supplement: FIG S1 [file mBio.00607-20-sf001.jpg]

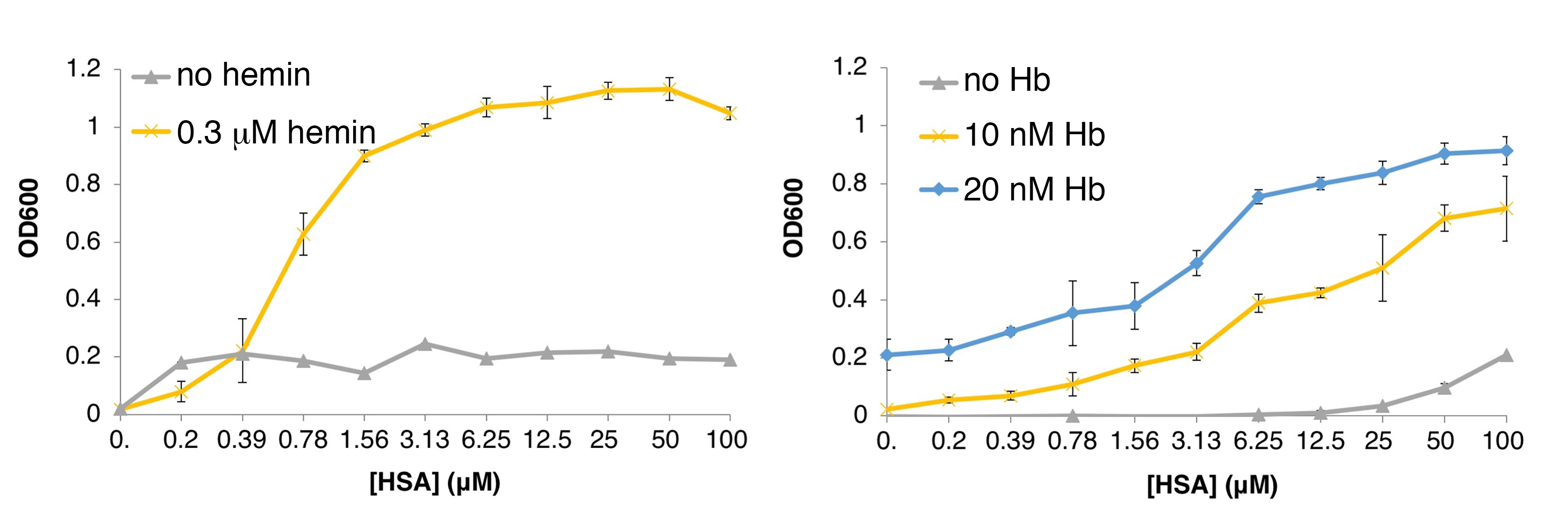

Supplement: FIG S2 [file mBio.00607-20-sf002.jpg]

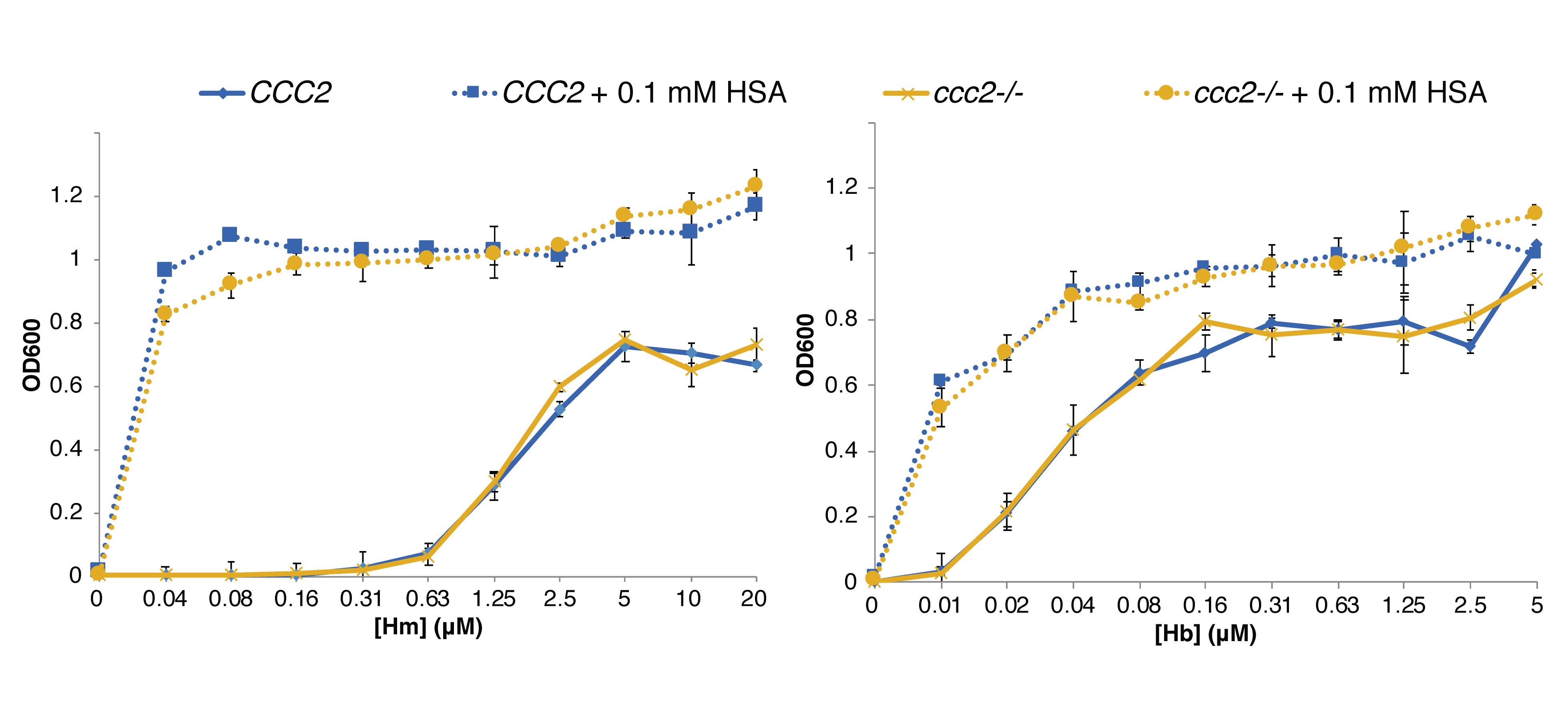

Supplement: FIG S3 [file mBio.00607-20-sf003.jpg]

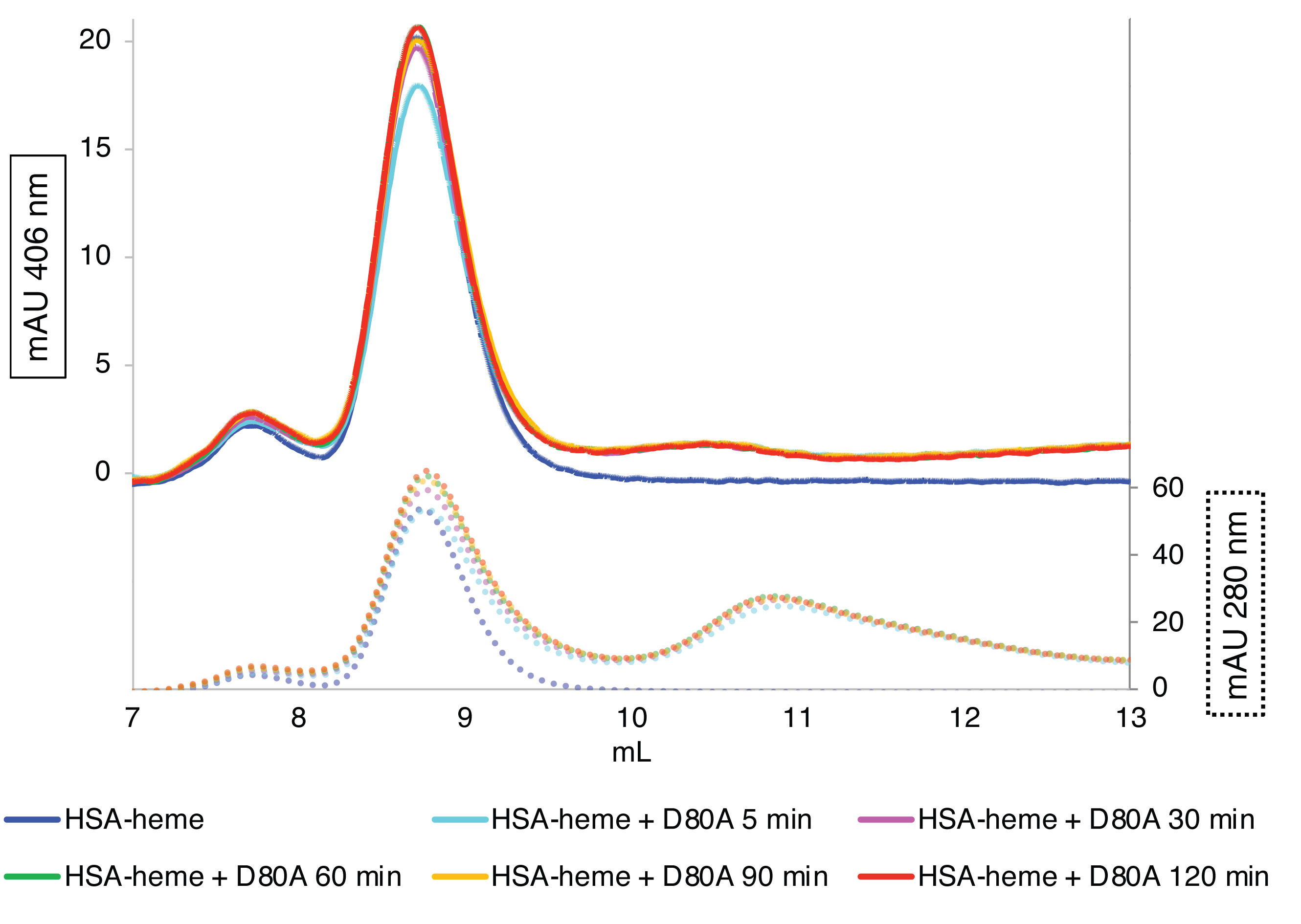

Supplement: FIG S4 [file mBio.00607-20-sf004.jpg]

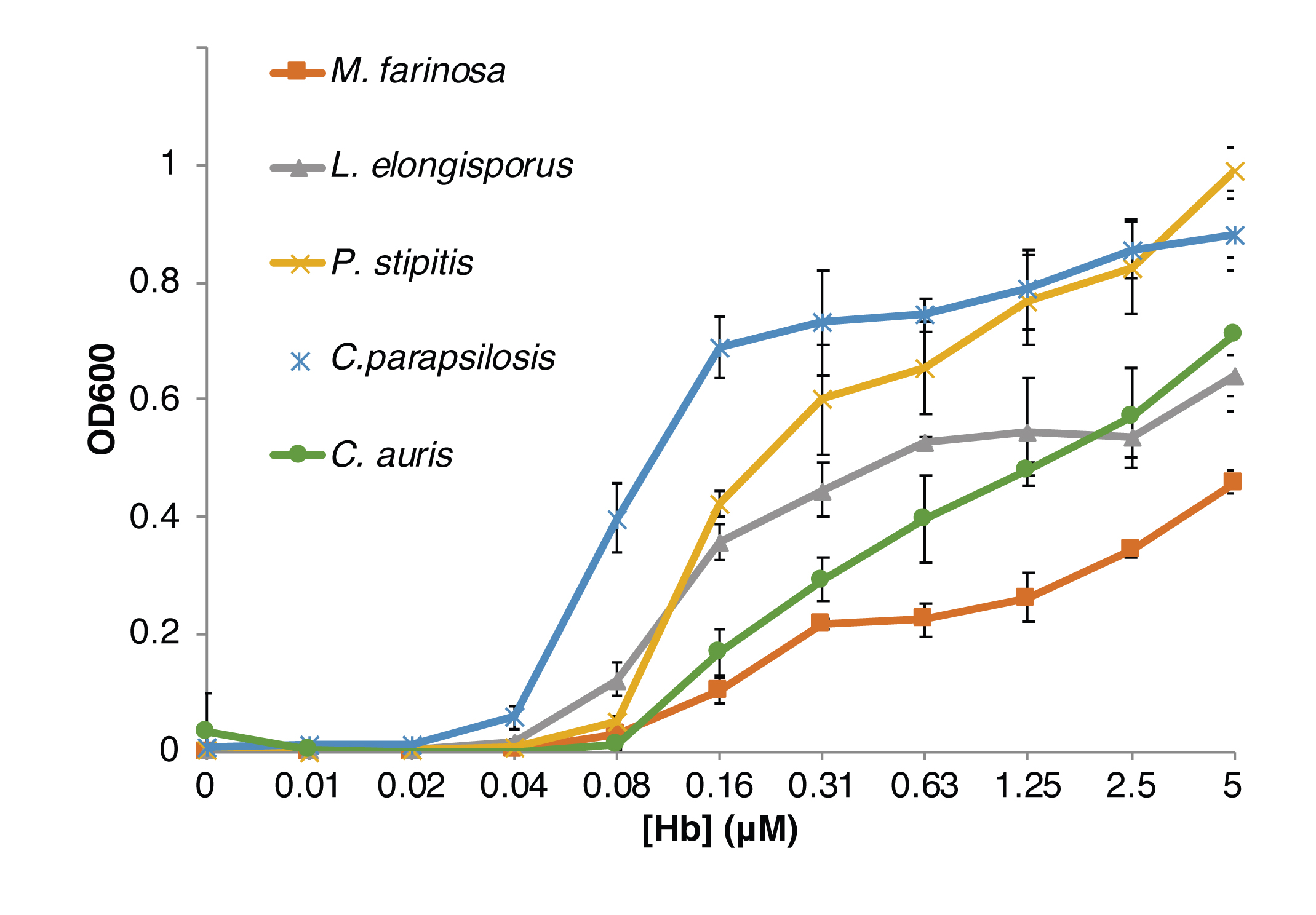

Supplement: FIG S5 [file mBio.00607-20-sf005.jpg]

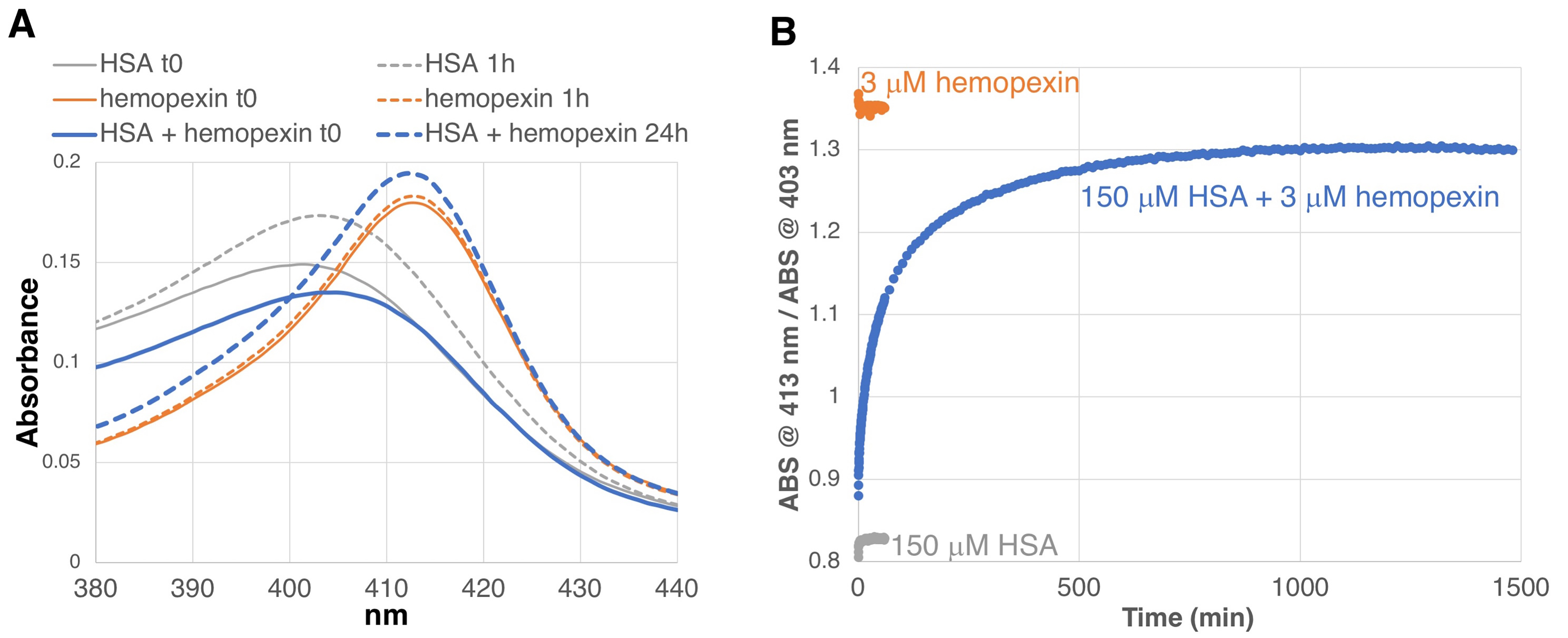

Supplement: FIG S6 [file mBio.00607-20-sf006.jpg]

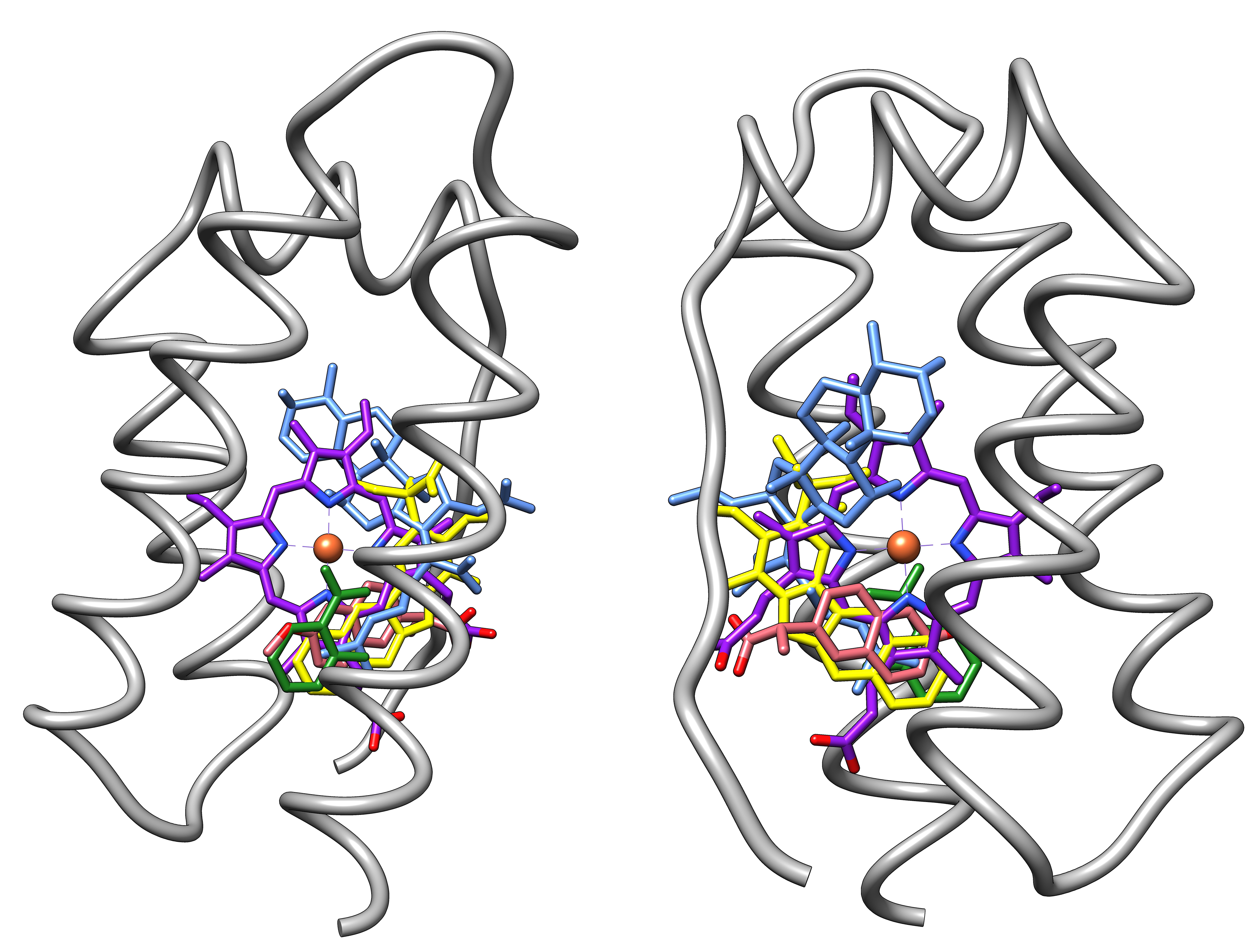

Supplement: FIG S7 [file mBio.00607-20-sf007.jpg]

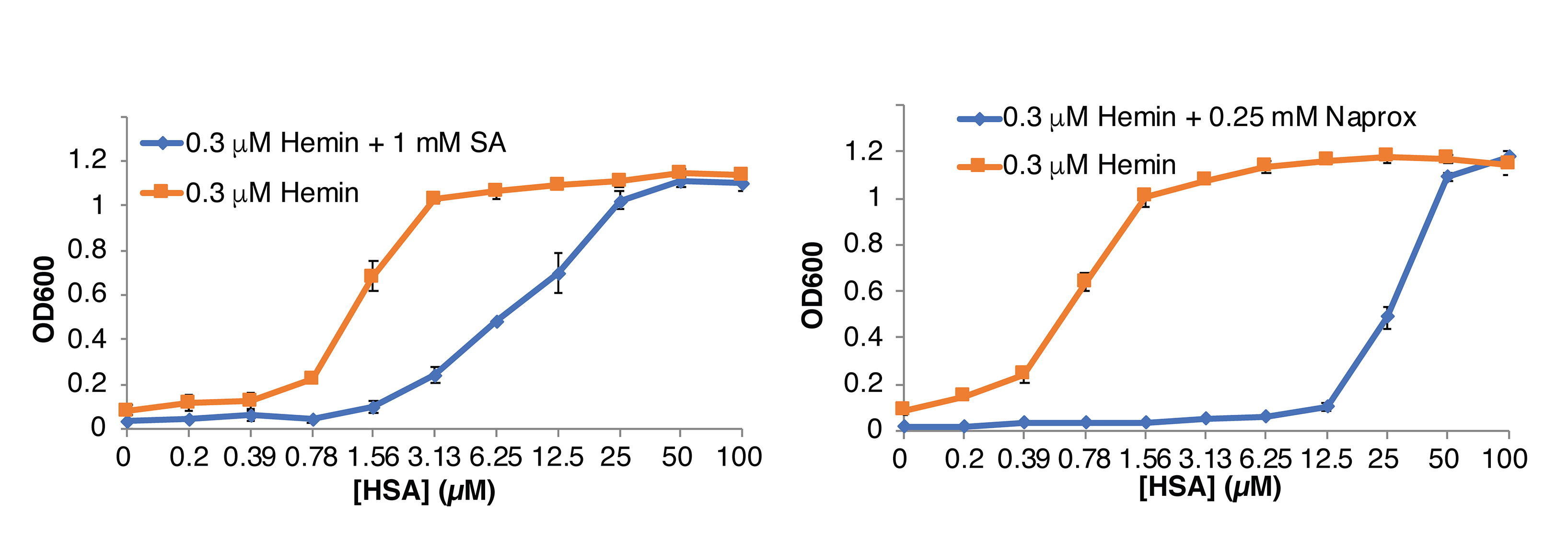

Supplement: FIG S8 [file mBio.00607-20-sf008.jpg]
