# Supplementary material for: Human Serum Albumin Facilitates Heme-Iron Utilization by Fungi
Source: mBio. 2020 Apr 21;11(2):e00607-20. doi: 10.1128/mBio.00607-20 (PMC7175094; doi:10.1128/mBio.00607-20)
Supplement: TABLE S1 [file mBio.00607-20-st001.pdf]

Table S1. Summary of the effect of HSA-binding drugs on *C. albicans* growth and on heme utilization stimulated by 5 μM HSA.

Growth was tested with the *C. albicans* KC2 (*CCC2*) strain in YPD medium + 1 mM ferrozine, and with the KC68 (*ccc2*$^{-/-}$) strain in the same medium supplemented with 3 μM hemin. HSA-heme utilization was tested in the KC68 background, in the same medium supplemented with 5 μM HSA and 0.3 μM hemin.

Drug	Minimal inhibitory concentration for HSA-heme utilization	Minimal inhibitory concentration for growth
Allosteric drugs		
Ibuprofen	-	4 mM
Warfarin	-	> 4 mM
Rifampicin	-	> 2 mM
Isoniazid	-	> 2 mM
Direct-competing drugs		
Salicylic acid	1 mM	5 mM
Naproxen	0.13 mM	2 mM
Camptothecin	-	> 0.15 mM
Fusidic acid	-	4 mM
