# Supplementary material for: Human Serum Albumin Facilitates Heme-Iron Utilization by Fungi
Source: mBio. 2020 Apr 21;11(2):e00607-20. doi: 10.1128/mBio.00607-20 (PMC7175094; doi:10.1128/mBio.00607-20)
Supplement: TABLE S2 [file mBio.00607-20-st002.pdf]

Table S2. List of fungal strains.

Name	Genotype / strain number	Origin
Candida albicans strains		
KC2 = CAI4	*ura3Δ::imm434/ura3Δ::imm434*	(1)
KC68	KC2 *ccc2Δ::hisG/ccc2Δ::hisG*	(2)
KC139	KC68 *rbt5Δ::hisG/rbt5Δ::hisG*	(3)
KC485	KC68 *pga7Δ::hisG/pga7Δ::hisG*	(4)
KC782	KC68 *csa2Δ::hisG/csa2Δ::hisG*	(5)
Other fungal species		
Candida parapsilosis	Y2315	ARS NRRL[a]
Candida auris	Clinical isolate	Tel-Aviv Sourasky medical center – Ronen Ben-Ami
Lodderomyces elongisporus	CBS 2605	CBS [b]
Meyerozima farinosa	CBS 185	CBS
Pichia stipitis	CBS 5773	CBS

[a] ARS NRRL = Agricultural Research Service culture collection, Peoria, IL

[b] CBS = Centraalbureau voor Schimmelcultures, Utrecht, The Netherlands.

SUPPLEMENTAL REFERENCES

1. Fonzi WA, Irwin MY. 1993. Isogenic strain construction and gene mapping in Candida albicans. Genetics 134:717–728.
2. Weissman Z, Shemer R, Kornitzer D. 2002. Deletion of the copper transporter CaCCC2 reveals two distinct pathways for iron acquisition in Candida albicans. Mol Microbiol 44:1551–1560.
3. Weissman Z, Kornitzer D. 2004. A family of Candida cell surface haem-binding proteins involved in haemin and haemoglobin-iron utilization. Mol Microbiol 53:1209–1220.
4. Kuznets G, Vigonsky E, Weissman Z, Lalli D, Gildor T, Kauffman SJ, Turano P, Becker J, Lewinson O, Kornitzer D. 2014. A Relay Network of Extracellular Heme-Binding Proteins Drives C. albicans Iron Acquisition from Hemoglobin. PLoS Pathog 10:e1004407.
5. Nasser L, Weissman Z, Pinsky M, Amartely H, Dvir H, Kornitzer D. 2016. Structural basis of haem-iron acquisition by fungal pathogens. Nat Microbiol 1:16156.
